# Antidepressants and Circadian Rhythm: Exploring Their Bidirectional Interaction for the Treatment of Depression

**DOI:** 10.3390/pharmaceutics13111975

**Published:** 2021-11-21

**Authors:** Soraia Silva, Joana Bicker, Amílcar Falcão, Ana Fortuna

**Affiliations:** 1 Laboratory of Pharmacology, Faculty of Pharmacy, University of Coimbra, Azinhaga Sta. Comba, 3000-548 Coimbra, Portugal; soraia.silva92@gmail.com (S.S.); acfalcao@ff.uc.pt (A.F.); anacfortuna@gmail.com (A.F.); 2 CIBIT—Coimbra Institute for Biomedical Imaging and Translational Research, University of Coimbra, Azinhaga Sta. Comba, 3000-548 Coimbra, Portugal

**Keywords:** antidepressant, circadian rhythm, chronopharmacokinetics, chrono-pharmacodynamics, chronotoxicology, depression, clinical studies

## Abstract

Scientific evidence that circadian rhythms affect pharmacokinetics and pharmacodynamics has highlighted the importance of drug dosing-time. Circadian oscillations alter drug absorption, distribution, metabolism, and excretion (ADME) as well as intracellular signaling systems, target molecules (e.g., receptors, transporters, and enzymes), and gene transcription. Although several antidepressant drugs are clinically available, less than 50% of depressed patients respond to first-line pharmacological treatments. Chronotherapeutic approaches to enhance the effectiveness of antidepressants are not completely known. Even so, experimental results found until this day suggest a positive influence of drug dosing-time on the efficacy of depression therapy. On the other hand, antidepressants have also demonstrated to modulate circadian rhythmicity and sleep–wake cycles. This review aims to evidence the potential of chronotherapy to improve the efficacy and/or safety of antidepressants. It includes pre-clinical and clinical studies that demonstrate the relevance of determining the most appropriate time of administration for antidepressant drugs. In parallel, their positive influence on the resynchronization of disrupted circadian rhythms is also herein discussed. It is expected that this review will promote the investigation of chronotherapy for the treatment of depression, contribute to a better understanding of the relationship between antidepressants and circadian rhythms, and consequently promote the development of new therapeutics.

## 1. Introduction

All organisms display biological processes with rhythmic oscillations of 24 h periodicity defined as circadian rhythms. Among them is the sleep–wake cycle associated with the sleep hormone melatonin, which has a 24 h-variation [1]. In a process regulated by noradrenergic and neuropeptidergic signaling, the pineal gland converts serotonin (5-hydroxytryptamine; 5-HT) to melatonin, which is then released into the systemic circulation [2]. Melatonin secretion is enhanced by darkness and inhibited by light, achieving plasma peak levels between 2h00 and 4h00 in the morning [2]. Other biological processes are repeated throughout the 24 h (i.e., ultradian rhythms) such as blood circulation, respiration, heart rate, and thermoregulation [3]. If repeated for periods longer than 24 h, rhythms are known as infradian, such as the menstrual cycle or seasonal rhythms [4].

At a molecular level, circadian rhythms are controlled by positive and negative feedback loops that dictate the transcription and translation of clock-genes [5]. The transcription factors, brain and muscle ARNT-like 1 (BMAL1) and circadian locomotor output cycles kaput (CLOCK), dimerize and bind to E-box or E-box-like elements of the promotor region of clock-genes, inducing the transcription of rhythmic clock genes Period 1 and 2 (*PER1* and *PER2*) and cryptochrome (*CRY*) [6]. In the nucleus, the corresponding expressed proteins PER and CRY inhibit BMAL1:CLOCK heterodimerization in a negative feedback loop. The PER and CRY display peak levels at the end of the day and decrease during the night, in opposition to BMAL1:CLOCK activity [6]. Simultaneously, a secondary mechanism mediated by retinoid-related orphan receptors (RORs) and reverse erythroblastosis virus α (REV-ERBα) induce and inhibit *BMAL1* transcription, respectively [5,7].

In mammals, the central clock is found in the suprachiasmatic nucleus (SCN) located in the medio-frontal hypothalamus. The SCN is responsible for maintaining all body cells synchronized by directly or indirectly adjusting peripheral clocks through the synthesis of hormones, such as melatonin or cortisol [5,8]. For instance, neuronal and hormonal clock outputs regulate cell growth, renal filtration, cognition, nutrient metabolism, and immune function [9]. Sunlight, temperature, or food intake are known time-givers (*zeitgebers* in German), i.e., external factors that modulate circadian rhythms [10]. Light signals are received by visual photoreceptors and retinal photosensitive ganglion cells, and the nerve impulse is then transmitted to the SCN by demyelinated axons via retinohypothalamic tract [5].

Dysregulation of circadian rhythms can lead to several disorders including heart diseases, diabetes, cancer, or depression [11]. Depression is a common mental disorder that affects over 300 million people of all ages [12]. The situation worsened in 2020 due to the COVID pandemic restrictions and an increase of 25% of cases has been reported worldwide [13]. In neuropsychiatric disorders such as depression, melatonin and cortisol are deregulated [14]. Indeed, in moderate and severely depressed patients, nocturnal salivary melatonin levels are 1.5- and 3.3-fold lower, respectively, than in healthy individuals, regardless of gender [15]. Melatonin is also associated with sleep–wake rhythms and it is known that bedtime melatonin concentrations have a negative correlation with the severity of depressive symptoms [16]. These results emphasize the use of melatonin as clinical biomarker and as a potential marker of treatment response. Moreover, 6-sulfatoxymelatonin, the main urinary melatonin metabolite, has been proposed as a biomarker for several antidepressant treatments [17,18]. On the other hand, patients diagnosed with major depressive disorder (MDD) show significantly higher waking salivary cortisol levels compared to healthy subjects [19]. In plasma of psychiatric patients, increased cortisol levels during inactive hours have been also associated with psychotic MDD [20].

Depression indicators such as concentration and memory deficit or slow reaction time, vary throughout the day, being more prominent in the morning than evening [21]. Findings from a meta-analysis revealed more severe mood symptoms and a higher likelihood of a mood disorder in individuals with an evening chronotype, who wake up and sleep at late hours and are more active at the end of the day [22,23]. Furthermore, evening-type individuals normally take a higher number of antidepressants with reduced efficacy but similar side effects, compared with morning chronotypes [24]. These data emphasize the importance of light–dark cycles in the development of mood disorders, including depression, and the use of individualized therapy for its treatment.

A deficient stimulation of post-synaptic neurons by norepinephrine (NE) and 5-HT is one of the principal factors underlying the physiopathology of depression [25]. The mechanism of action of most antidepressant drugs relies on slowing the reuptake and thus raising the concentration of those neurotransmitters in the synaptic cleft, increasing neurotransmission and the relief of depressive symptoms [26]. The classification of antidepressant drugs is associated with their respective mechanism of action: selective 5-HT reuptake inhibitors (SSRIs), 5-HT and NE reuptake inhibitors (SNRIs), tricyclic antidepressants (TCAs), monoamine oxidase inhibitors (MAOIs), and atypical antidepressants [27]. New classifications have been proposed by regional and international organizations, including European, Asian, American, and International Colleges of Neuropsychopharmacology and International Union of Basic and Clinical Pharmacology. These classifications rely on a pharmacologically-driven nomenclature focusing on approved indications, efficacy, side effects, and neurobiology, but until today, none have been unanimously accepted [28,29]. Antidepressants are usually applied in chronic treatments for long periods of time, despite revealing several side effects. In particular, TCAs exhibit poor tolerability and adverse effects, such as dry mouth, tremors, blurred vision, body weight gain, memory disorders, postural hypotension, and gastrointestinal disturbances and sedation, which ultimately undermine adherence to treatment [30,31]. Since depression is usually worse in the morning, antidepressants can be administered in this period, although their side effects may shift dosing to bedtime [32]. Even so, less than 50% of depressed patients achieve remission following several pharmacological interventions.

Based on the aforementioned bidirectional interactions between circadian rhythms and depression, interest in chronotherapy with antidepressants has increased exponentially [33]. Pharmacokinetic processes, specifically, absorption, distribution, metabolism, and excretion (ADME), present time-dependent oscillations that can lead to different concentrations in the plasma and tissues and, therefore, distinct therapeutic effects [34]. Complementarily, pharmacodynamic studies describe the association between drug concentrations and their effects [35]. The role of clock genes on antidepressant targets seem to influence antidepressant efficacy and side effects during the day [36,37].

Chronopharmacokinetics, chrono-pharmacodynamics, and chronotoxicology are hence defined by differences in pharmacokinetics and pharmacodynamics due to biological rhythms in ADME or in therapeutic and toxic effects, respectively [38,39]. Variations with clinical impact must be considered to adjust the dosing-time of an antidepressant, in order to improve its benefits [40]. According to these observations, defining the best dosing-time for antidepressant administration may result in better pharmacokinetic profiles, improved therapeutic effect and less toxicity. Hence, this review highlights clinical and non-clinical investigations regarding the chronopharmacokinetics and chrono-pharmacodynamics of antidepressant drugs, since understanding their chronopharmacological profiles may improve depression treatment. To finalize, the influence of antidepressants on the modulation of circadian rhythmicity will also be herein explored, in order to provide a better insight of its clinical importance.

For this review, databases including PubMed and Google Scholar were searched for papers using the terms “circadian AND rhythm AND antidepressants”, “chronopharmacology OR chronopharmacokinetics OR sleep AND antidepressants”. Due to the scarcity of data, no restrictions of time period were applied. Additionally, a search in ClinicalTrials.gov for ongoing clinical trials was performed (registered until 30 September 2021), but no results were found with the terms “chronopharmacokinetic OR chronopharmacology OR chronotoxicology AND depression”.

## 2. Pharmacokinetics of Antidepressants

### 2.1. Circadian Rhythm Effect on Pharmacokinetic Stages

#### 2.1.1. Absorption

**Figure 1:**
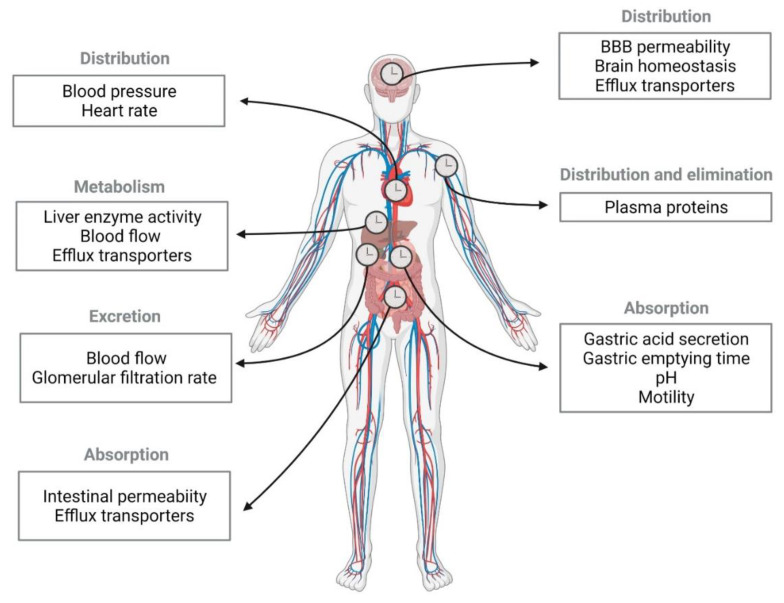
Physiological processes regulated by circadian rhythms with strong effect on the pharmacokinetics of antidepressants. BBB, blood–brain barrier.

Chronopharmacological studies with antidepressants are often performed *in vivo*. Light is a strong *zeitgeber* in rodents, which display an active phase during the night. For this reason, studies are usually performed under a 12h00 light–12h00 dark cycle, during which drugs are administered at different *zeitgeber* times (ZT). The ZT0 corresponds to the moment that lights are turned on and ZT12 when lights are turned off [41]. In contrast, chronotherapy in humans needs to consider *zeitgeber* factors other than light, namely mealtime, oxygen levels, temperature, and exercise [41]. Pharmacokinetic alterations at different drug dosing times are associated with intrinsic circadian rhythms in several tissues that are also involved in physiological ADME (Figure 1). Chronopharmacokinetic parameters of antidepressants have mostly been studied for TCAs in rodents (Table 1) and humans (Table 2), probably due to their narrow therapeutic index.

Since antidepressants are orally administered, their bioavailability depends not only on their physicochemical properties, such as lipophilicity, but also on physiological processes of the digestive system modulated by circadian rhythms that may be disrupted in depressed patients [42]. As previously stated, food is a strong zeitgeber, but only when its availability is limited during the day, leading mammals to adjust their daily rhythms and behavior [43]. Pre-mealtime increases core temperature, corticosterone levels, and duodenal disaccharidases production [43]. In turn, food intake influences gastric acid secretion, pH, motility, and gastric emptying time, all of which, together with food–drug interactions, can affect drug release and absorption [44].

Although the mechanism of action of trimipramine is not fully characterized, it is known to be a TCA that differs from others, particularly because it does not directly inhibit reuptake transporters, namely the 5-HT transporter (SERT) or NE reuptake transporter (NET) [45]. Chronopharmacokinetic studies in humans treated with two oral formulations of the trimipramine (solution and tablet) showed that tablets seem to be less affected by dosing-time (Table 2) [46]. Importantly, drug absorption remains faster after solution administration than tablets administration. Solutions demonstrated significantly higher maximum concentration (C_max_), lower time to reach C_max_ (t_max_), and lower mean residence time (MRT) in the morning (under fasting conditions) than at night (after a meal). The authors refer that food delays and reduces the intestinal absorption of solutions and, therefore, different pharmacokinetic results may be found in fasting or after a meal. In contrast, tablet formulations have similar pharmacokinetic parameters in plasma, independent of the administration time [46]. Likewise, the single dose administration of sertraline tablets, a SSRI antidepressant, revealed that the drug bioavailability is not influenced by the time of administration (Table 2) or by the presence of food [47].

Intestinal permeability shows daily rhythms due to variations of expression of tight junction proteins that regulate the epithelial paracellular pathway [51]. In the small intestine, mRNA levels of occludin and claudin-3 were higher during the late dark phase than the late light phase and inversely associated with paracellular permeability data [52]. A study performed by Oh-Oka et al. reported that, in the large intestine of mice, BMAL1 and CLOCK bind to the E-box element in the promoter regions of occludin and claudin-1 genes and affect their transcriptional responses [51]. Colonic permeability was higher at the beginning of the dark phase (ZT16) than during the light phase (ZT4) and inversely related with occludin and claudin-1 expression levels [51]. Meanwhile, the disruption of circadian rhythms by light–dark shifting results in a lower expression of zonula occludens-1 and in colon hyperpermeability [53]. It has also been observed that patients with MDD may have a leaky gut that activates their inflammatory response system. This reaction plays a critical role in the pathophysiology of depression because it increases the production of pro-inflammatory cytokines and weakens cellular immunity [54].

From another point of view, it is important to bear in mind that several antidepressant drugs are known as substrates of efflux transmembrane ATP-binding cassette (ABC) proteins, including P-glycoprotein (P-gp; ABCB1; MDR1) [55,56,57,58,59] and Breast Cancer Resistant Protein (BCRP; ABCG2) [60]. These transporters are expressed in several tissues, namely the intestine, kidney, liver, and blood–brain barrier (BBB). They reduce the bioavailability, facilitate the elimination, and hamper the access of compounds to the brain, including antidepressant drugs [61]. The expression of P-gp in the intestine is modulated by proline- and acid-rich basic leucine zipper (PAR bZIP) proteins, particularly hepatic leukemia factor (HLF), whose expression is regulated by core oscillator components [62]. The HLF and E4 promoter binding protein-4 (E4BP4), a putative antagonist of PAR bZIP proteins, respectively, increase and decrease the mRNA levels and expression of P-gp [63]. In the mouse intestine, *Mdr1a* mRNA levels exhibit a significant 24 h daily variation, increasing during the light phase with a peak at ZT12, when the lights go off [63,64]. Similarly, in the rat jejunum, P-gp mRNA varies 5.4-fold with the circadian time [65]. Consistent with the daily rhythmicity observed in total protein with a peak level at ZT8, the P-gp function in the intestine is significantly higher at ZT12 than ZT0 [64]. Additionally, feeding patterns and gender also influence the expression and activities of the ABC transporters during the day, since circadian amplitudes of mRNA and protein levels of P-gp in the ileum are larger in female mice than in male mice [66]. A study performed with cynomolgus monkeys, diurnally active animals, reported that the intestinal expression of *ABCB1* mRNA in monkeys oscillated in the opposite phase of rodents. However, the intestinal expression and function of P-gp was similar between both species [67]. The authors justified the delayed mRNA oscillation in monkeys due to slower protein synthesis and/or transportation into the intestinal membrane [67]. These results suggest that the data generated from mouse models may be transferable to humans, provided that the P-gp protein levels, and not exclusively mRNA levels, are taken into consideration.

Multiple-dose experiments provide a better insight of the pharmacokinetics of antidepressants administered to humans. Amitriptyline is a TCA and a P-gp substrate [68]. Similar pharmacokinetic parameters were found in plasma between administrations at ZT4 or ZT16 after multiple dosing in rats, but the absorption rate constant (k_a_) was 1.46 higher in the active phase than in the rest phase (Table 1) [48]. In humans, the absorption of amitriptyline also presents a daily rhythm [37]. Oral administrations of amitriptyline hydrochloride (50 mg) in the morning (9h00) or evening (21h00) for three weeks (Table 2) demonstrated that t_max_ in plasma was significantly shorter with morning doses (3.2 h vs 4.4 h) while k_a_ values were higher (0.36 h^−1^ vs 0.25 h^−1^) [37]. The active metabolite of amitriptyline, nortriptyline, also had a faster absorption after morning administration than in the evening, although the difference was not statically significant (Table 2) [50].

Similarly to P-gp, BCRP expression demonstrates daily oscillations in the intestine of mice. It is induced through the activation of transcription factor 4 (ATF4), a molecule regulated by clock genes [69,70]. In the rat jejunum, BCRP mRNA levels vary slightly (1.6-fold) with the circadian time [65]. The BCRP mRNA levels in the mouse intestine increase during the light phase, with peak values near its end (between ZT6 and ZT10). Protein expression levels and function are higher in the beginning of the dark phase (ZT14) [70]. In agreement, BCRP protein levels in the small intestine of monkeys are higher between 15h00 and 21h00 [67].

The aforementioned results regarding P-gp and BCRP in the intestine suggest that their substrates may have higher bioavailability if administered in the morning, when the expression levels of efflux transporters are lower. Although sertraline is a known BCRP substrate [60], there are no studies in literature concerning the influence of the circadian BCRP oscillations on its intestinal absorption.

#### 2.1.2. Distribution

The cardiovascular system is susceptible to circadian rhythmicity since blood pressure, heart rate, and plasma protein levels display daily oscillations [71,72]. The blood pressure of mammals has a 24 h cycle, with a peak during their active phase and a 10–20% slope at rest phase, along with a 17% daily fluctuation of total plasma proteins [73,74]. Particularly for antidepressant drugs that are highly protein-bound, distribution is strongly affected by the presence of plasma proteins, mainly albumin and α_1_-acid glycoprotein [75,76]. A study performed in rats demonstrated that plasma protein levels are higher in the active phase [74]. Furthermore, in healthy humans, α_1_-acid glycoprotein plasma levels are also increased during the active phase [77]. Hence, these protein fluctuations may be important to adjust the dosing-time of highly protein-bound antidepressants since differences of unbound fraction and tissue exposure can affect drug efficacy and toxicity [48]. A single intragastric administration of amitriptyline in rats (64 mg/kg) at six different time-points revealed highest bioavailability at the end of the active phase (ZT22) [48]. In fact, the total drug exposure was higher in the beginning of the rest phase, even though the k_a_ after intragastric administration was unaltered during the light–dark cycle [48]. Exposure in liver and lung, given by area under the concentration–time curve (AUC), showed a 24 h oscillation (peak at ZT4). Moreover, multi-dosing for 10 days demonstrated significantly higher AUC values in liver, lung, and kidney tissues in the light than in the dark phase [48]. These results may be linked to the reduction of plasma protein levels during the light phase, since amitriptyline is a highly protein-bound drug (95%) [78]. In another study, the concentrations of imipramine and its active metabolite, desipramine, were evaluated in rat plasma after a single injection, at two different time-points [49]. Desipramine revealed a lower t_max_ and faster distribution, given by higher distribution half-life time (t_1/2α_) values at ZT0.5 than those at ZT12.5 (Table 1). Indeed, the plasma exposure of desipramine, after oral chronic treatment with imipramine (15 mg/kg) for 14 days, revealed a 24 h oscillation, with the highest concentration detected at ZT7 (48.9 mg/L) and the lowest at ZT13 (12.4 mg/L) [49].

The therapeutic targets of antidepressant drugs are located in the central nervous system (CNS), whose access is limited by blood-CNS barriers, including the BBB and the blood-cerebrospinal fluid (CSF) barrier. Circadian rhythms have an important role in BBB homeostasis and integrity, since the deletion of the clock component BMAL1 leads to BBB hyperpermeability [79]. Additionally, a strong circadian gene expression of *Per2* in the choroid plexus is responsible for the adjustment of the SCN clock and brain homeostasis through the CSF [80]. Sleep–awake cycles also influence the volume of the interstitial space. Xie et al. evidenced that natural sleep and ketamine/xylazine anesthesia increase CSF influx and, therefore, the clearance of neural metabolites from the brain of mice [81].

One of the main causes behind antidepressant inefficacy is the overexpression of P-gp and BCRP by the endothelial cells of BBB of depressed patients [61] and the efflux of xenobiotics across the BBB of mammals is known to be regulated by circadian rhythms [82]. Nonetheless, there is not much data concerning diurnal variations in the transcript and protein levels of efflux transporters in the brain and the studies performed until today are conflicting. Pulido et al. observed an inverse association between P-gp activity and the active phase of wild-type mice [83]. The authors reported a diurnal oscillation of P-gp transcription (peak at ZT12) in the brain, which increased during the light phase and decreased during the dark phase. It was modulated by PAR bZip transcription factors, specifically albumin D box-binding protein (DBP), thyrotroph embryonic factor (TEF), and HLF [83]. Accordingly, the accumulation of a P-gp substrate (rhodamine-123) in the brain was higher in the beginning of the light phase [83]. In opposition, Zhang et al. did not find circadian alterations of mRNA or protein levels of P-gp in the BBB of mice or humans [82]. However, its activity was modulated by clock genes. In mice, higher efflux activity was observed during the active phase, but transcription levels and protein expression were unaffected. The magnesium transporter transient receptor potential cation channel, subfamily M, member 7 (TRPM7) contributed to efflux rhythms in human cultured brain endothelial cells [82]. The BMAL1 modulates transcript and protein expression of TRPM7 during active periods, which enhances intracellular magnesium levels and promotes efflux activity. It was observed that BBB permeability in *Drosophila* is regulated by 24 h cyclically expressed gap junctions [84]. During the rest phase, increased gap junctions reduce intracellular magnesium and, therefore, lead to higher brain exposure at night [84]. A study performed by Kervezee et al. with resort to intracerebral microdialysis demonstrated that the exposure of a P-gp substrate in rat brain tissue displayed a diurnal rhythm, no longer observed after P-gp inhibition [85]. The authors verified that drug accumulation in the rat brain is higher during the rest phase than active phase [85]. In contrast, Savolainen et al. found that the highest brain uptake of a radiolabeled P-gp substrate, [^18^F]MC225, during the active phase (ZT15), significantly decreased until ZT21 [86]. Still, the role of P-gp in these results was not directly evaluated, since the effect of P-pg inhibition was not assessed. Interestingly, intragastric administrations of amitriptyline in rats revealed a 12 h oscillation of drug levels in the brain (peaks at ZT4 and ZT16) [48]. It is known that P-gp expression in the BBB has the potential to reduce amitriptyline concentrations in the brain [87,88], however, other factors such as clearance and daily oscillations of plasma protein levels may have also influenced the brain exposure of this drug.

Imipramine and desipramine levels in the rat brain were analyzed after administrations at different times of the light–dark cycle [49]. Their C_max_ and AUC in the brain were 1.6-fold higher in the light phase (ZT12.5) than in the dark one (ZT0.5), following intravenous or intraperitoneal single administrations (Table 1). The increase of these values could be related with P-gp activity, since both drugs accumulate in the brain after P-gp inhibition with verapamil [89]. Furthermore, the chronic administration of imipramine (15 mg/kg) in drinking water for two weeks caused concentration variations of imipramine and its metabolite in the brain (more than 3-fold within 24 h). Peak levels were reached at ZT13 (2.81 mg/L) and ZT7 (0.67 mg/L), respectively, suggesting that both undergo time-dependent oscillations. Curiously, lower concentrations were detected at ZT7 (0.83 mg/L) for imipramine and ZT13 (0.20 mg/L) for desipramine. Nonetheless, it is important to refer that imipramine altered the drinking behavior of rats, since higher drinking volumes were observed at ZT12-ZT14, compared with those of the control group [49]. This may have been caused by the adverse effect of imipramine, i.e., xerostomia.

Transcript levels of BCRP in the BBB display circadian oscillations with peak levels at ZT14, independent of BMAL1 [82]. To the best of our knowledge, data on daily oscillations of the BCRP protein and function on the BBB remain scarce. Additional studies would assist our current understanding of the impact of circadian rhythms on the xenobiotic efflux in the BBB.

#### 2.1.3. Metabolism and Excretion

Liver enzyme activity and hepatic blood flow are two time-dependent processes that drive hepatic drug metabolism [90]. Lipophilic drugs, such as antidepressants, are metabolized in the liver into more hydrophilic polar metabolites in three phases [91]. Phase I functionalization reactions are mostly performed by the cytochrome P450 (CYP) enzyme superfamily, specifically the isoforms CYP1A2, CYP2C9, CYP2C19, CYP2D6, and CYP3A4 [91]. After phase II conjugation reactions, metabolites may be excreted into the bile or transported into the systemic circulation by efflux transporters (phase III). The PAR bZip and an atypical nuclear receptor expressed abundantly in the liver, named as small heterodimer partner (SHP), play a pivotal role in CYP activity 24 h oscillations in mice, controlling daily variations of detoxification and drug metabolism [62,92]. In addition, in mice, Zhang et al. described that the mRNA levels of phase I enzymes increase in the dark phase, while phase II enzymes rise in the light phase [93]. Analyzing serum-shocked cells of a human hepatocellular carcinoma cell line (HepG2), a 24 h oscillation in the expression of CYP3A4 and CYP2D6 was revealed [94,95]. Transcript levels seem to be considerably more affected than protein levels when circadian rhythms are disrupted. The rhythmicity of liver proteins can be explained by rhythmic mRNAs, and translational and post-translational regulation and feeding behavior [96].

Regarding efflux transporters, Ando et al. reported time-dependent mRNA levels of hepatic P-gp in mice that increase in the light phase and peak at ZT16 [64]. Still, total P-gp protein levels were constant throughout the light–dark cycle in mice [64] and similar results were found in monkeys [67]. Recent investigations confirmed that P-gp mRNA levels vary with time in mouse liver, but protein levels present only a slight daily oscillation [97]. This discrepancy between mRNA and protein levels can be explained by several factors such as post-transcriptional mechanisms, oscillations of protein degradation rate, or experimental conditions that require further investigation. Concerning BCRP, mRNA and protein levels do not vary during the light–dark cycle in the liver of mice or monkeys [67,97], although transcript levels of isoform *Abcg2* containing exon 1B oscillated in mouse liver [70]. Nevertheless, in zebrafish, active during the light phase, hepatic *Abcg2* mRNA levels were higher in the dark phase (peak at ZT18) than in the light phase [98]. Consequently, additional studies are needed to clarify the extent of rhythmic expression and function of efflux transporters in the liver.

Metabolic oscillations appear to have a weaker role in the chronopharmacokinetics of antidepressants. For instance, the elimination half-life time (t_1/2β_) of sertraline did not change after administration to humans in the morning or evening (Table 2) [46]. Similarly, the administration of imipramine to rats revealed that t_1/2_ was not time-dependent (Table 1) [49]. Moreover, imipramine versus desipramine exposure ratios in plasma were 1.7 and 1.8 during the light and dark phase, respectively, suggesting a constant metabolic process [49]. The lack of data concerning the influence of circadian rhythms on the metabolism of antidepressants reveals that additional studies in this area are warranted.

The excretion of antidepressants and their metabolites is predominantly renal and involves three processes: glomerular filtration, active tubular secretion, and tubular reabsorption [99]. In a mouse kidney, P-gp mRNA and protein expression do not appear to present 24 h oscillations [64]. Nevertheless, daily variations of blood flow may explain clearance oscillations observed in the excretion of some drugs [100,101]. Single and daily administrations of amitriptyline for 10 days in rats showed significantly higher concentrations at ZT4 in the liver and kidney, compared to administrations at ZT16 [48]. This lower drug exposure was associated with clearance oscillations, since the highest clearance values were obtained between ZT13 and ZT16. In fact, its chronic administration revealed a higher clearance at ZT16 (13.61 L/h/Kg) than ZT4 (11.78 L/h/Kg). Identically, a lower t_1/2β_ was detected at ZT16 (2.90 h) compared with ZT4 (3.88 h) (Table 1) [48].

Glomerular filtration rates in rodents are higher during the dark phase, leading to higher urine volume [102]. Therefore, if drug administration is performed in the active phase when clearance is the highest, nephrotoxicity can be reduced [103]. More chronopharmacokinetic studies will improve the current knowledge about the influence of time on other classes of antidepressants since their elimination profiles were not sufficiently explored.

## 3. Pharmacodynamics of Antidepressants

### 3.1. Circadian Rhythm Effect on Antidepressant Drug Targets

**Figure 2:**
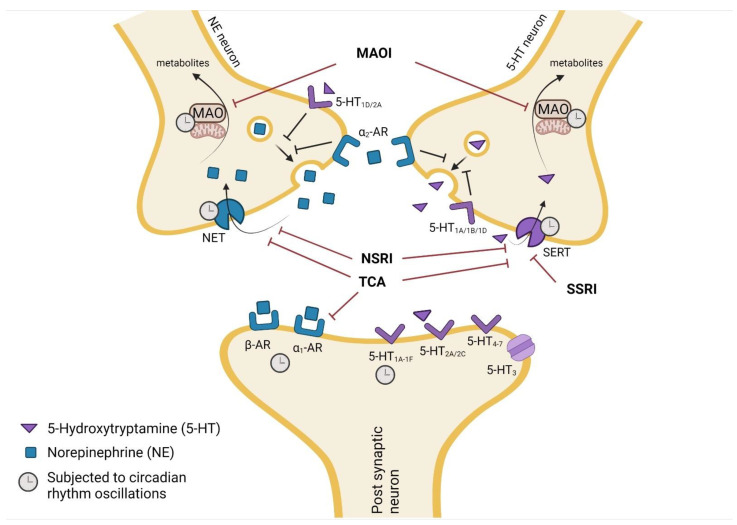
Summary of the mechanism of action of SSRIs, SNRIs, TCAs, and MAOIs at noradrenergic (left) and serotonergic (right) neurons. The influence of circadian rhythms on antidepressant targets is also depicted. SSRIs, SNRIs, and TCAs increase 5-HT neurotransmission through the direct blockade of SERT at presynaptic terminals. NET is inhibited by SNRIs and TCAs in noradrenergic neurons. MAOIs inhibit MAO enzymes present in mitochondria, responsible for breaking down neurotransmitters, such as 5-HT and NE. These processes increase the levels of 5-HT and NE in the synaptic cleft, leading to an antidepressant effect [26]. Circadian rhythms are known to affect the expression or activity of NET and SERT [104,105,106], 5-HT_1A_ receptor [106], adrenergic receptors [107], and MAO [108]. 5-HT_X_, 5-HT receptor subtypes; α- and β-AR, adrenergic receptors; MAO, monoamine oxidase; MAOI, MAO inhibitors; NET, NE transporter; SERT, 5-HT transporter; SNRI, SERT, and NET inhibitor; SSRI, SERT inhibitor; TCA, tricyclic antidepressant.

Different results of chronopharmacodynamic studies with antidepressants can be related with fluctuations of the pharmacokinetic parameters discussed in Section 2.1 or daily variations of the expression of antidepressant drug targets affected by circadian rhythms (Figure 2). Distinct mechanisms of action of antidepressant drugs may lead to different chronopharmacological profiles, and dosing-time can influence both therapeutic and toxic effects.

The monoaminergic hypothesis, established in 1965, postulates that depression is linked with noradrenergic and serotoninergic dysfunction in the CNS [109]. Hence, the development of antidepressants aimed towards the direct inhibition of SERT occurs, a member of the Na^+^/Cl^−^-dependent transporter family, or the blockade of both SERT and NET. The first is mediated by SSRIs, SNRIs, and TCAs and the second by SNRIs and TCAs. Notwithstanding, antidepressants exhibit different inhibitory potencies on reuptake transporters. The fact that inhibition is not equal among all drugs that prevent SERT and NET activity leads to different pharmacodynamic results in the same antidepressant classes [110].

Serotonergic and noradrenergic systems from the prefrontal cortex and hippocampus are very important to reduce depressive symptoms. Both systems have a 30% amplitude of mean content levels in the rat brain during a light–dark cycle [104]. The SERT transcription levels and activity revealed significant time-dependent changes in the mouse mid-brain, with higher levels during the active phase [105]. The 5-HT peak levels in the synaptic cleft occur at the end of the dark phase and are higher throughout the light phase than the dark phase. In the rat hippocampus, 5-HT turnover shows a peak during the ZT18–ZT22 and a trough at ZT10-14, while the NE turnover peaks between ZT22 and ZT2. Base levels were detected at ZT14-ZT18 [104]. Discrepancies of peak activity times in serotonergic and noradrenergic systems may be responsible for differences in the chronopharmacological profiles of antidepressants. In humans, positron emission tomography was applied to examine changes of the 5-HT_1A_ receptor and SERT in the brain of 40–56 healthy volunteers [106]. This study showed an increase and decrease of the 5-HT_1A_ receptor and SERT, respectively, in the midbrain during the day [106]. The increase of the 5-HT_1A_ receptor is directly correlated with the duration of daylight, leading to seasonal differences [106]. Indeed, potential binding values to SERT in the brain were significantly higher in the fall and winter, compared to the spring and summer, revealing a negative correlation with the daily amount of sunshine [111].

The TCAs are potent inhibitors of 5-HT and NE reuptake by binding to sodium-dependent transporters. They also block histamine H_1_ receptors, α_1_-adrenergic receptors, and muscarinic receptors causing sedative, hypotensive, and anticholinergic effects (e.g., blurred vision, dry mouth, constipation, urinary retention), respectively [112]. These receptors have been described to be modulated by circadian rhythm in rodents with higher expression during the day [107,113]. In the rat forebrain, the number of muscarinic receptors was significantly higher during the light than dark phase [113]. Likewise, the density of adrenergic receptors in the pineal gland of Syrian hamsters is higher during the light phase and decreases at the time of the peak of nocturnal melatonin production [107]. Activation of post-synaptic α_1_-adrenergic receptors is known to induce antidepressant effects, since NE exerts its function by binding to these receptors [114]. Nevertheless, their inhibition by TCAs may reduce the antidepressant effect of the noradrenergic system [115].

Monoamine oxidase (MAO) enzymes are located in the outer membrane of the mitochondria and catalyze the oxidation of monoamines, mediating the degradation of 5-HT and NE. These enzymes are the principal target of MAOI antidepressants. In the rat brain, MAO activity presents a 24 h oscillation, with higher levels during the light phase [108]. *In vitro* studies revealed that the transcription of the MAO-A promoter is regulated by clock components, namely BMAL1 and PER2 [108]. The direct binding of BMAL1 to the promoter of MAO-A in the rat brain is significantly higher during the light phase (ZT6) than the dark phase (ZT18). Moreover, *Per2* mutant mice showed no circadian oscillations of MAO-A expression and activity during the light–dark cycle [108].

Other neuronal systems, namely the dopaminergic and glutamatergic systems, also play an important role in the action of bupropion, an atypical antidepressant and dopamine reuptake inhibitor [116]. Dopamine, glutamate, and γ-aminobutyric acid (GABA) levels in the striatum and *nucleus accumbens* of the rat brain exhibit circadian oscillations and reach maximum levels during the dark phase [117]. Castañeda et al. analyzed the levels of these neurotransmitters during light–dark, full-dark, or full-light cycles. In the striatum region, only dopamine was influenced by light, whereas all neurotransmitters showed daily oscillations in the *nucleus accumbens* but were not regulated by light. The authors suggested that an endogenous mechanism could be responsible for this circadian variation [117].

Overall, several antidepressant targets are known to be regulated by circadian rhythms, which may have important implications regarding their efficacy at different dosing times. Nevertheless, much is yet unknown about this relationship. Further research in this field would benefit the design of future therapeutic approaches and improve the efficacy of currently available options.

#### 3.1.1. Animal Studies

Before chronopharmacological studies, it is important to ensure the entrainment of circadian rhythms in healthy mice to a standard light/dark cycle. Phenotyping circadian rhythms in mice requires measurements of their activity in free running conditions [118], quantifying circadian clock or clock-related genes [119], or monitoring sleep behavior [120]. Most animal studies regarding chronopharmacology for depression have been performed in animals without a depressive phenotype. However, it is important to consider the differences between rodents and humans and between normal and depressive conditions, bearing in mind that circadian rhythms are disrupted in depression. Thus, the use of healthy animals may complicate the transferability to clinical studies. To reduce the gap, mice can be induced into a depression-like state by chronic mild stress (CMS) or prolonged physical or social stress environments [121]. In addition, knockdown of clock gene *Bmal1* in mouse SCN is a validated model of depression and sufficient to cause helplessness, behavioral despair, and anxiety-like behavior in C57BL/6J mice [122].

The most common behavioral tests in rodents to evaluate antidepressant effects are the forced swimming test (FST) and the tail suspension test (TST). Both present high predictive reliability and validity but different sensitivity [123,124]. In TST, rodents are hung by the tail and failed efforts to escape lead to immobility. Pretreatment with antidepressant drugs decreases the duration of immobility. Generally, TST is a more consistent and sensitive model to detect SSRI activity than the traditional FST [124]. Results depend on mouse strain differences concerning NET and SERT binding levels, which lead to distinct immobility time in FST and TST [125,126]. Moreover, different immobility definitions are evaluated in these tests. Immobility in TST is the inability to maintain effort, while in FST, immobility is the reduction of movement to the minimum necessary to maintain the head above water [124]. Antagonism and genetic knockout of the GABA_B_ receptor, associated with anxiety and depressive symptoms, result in an antidepressant-like effect in FST, but not in TST [127]. During FST, rodents are placed in a cylinder tank with water and their immobility is evaluated. Even in single dose, rodents show decreased immobility during FST, representative of an antidepressant effect. In the modified FST (increase of water depth from 15–18 cm to 30 cm) it becomes possible to distinguish the serotonergic and noradrenergic activities of antidepressants by evaluating their immobility, swimming, and climbing [128].

Specifically, NET inhibition increases climbing, whereas inhibition of SERT selectively increases swimming [129]. However, for a reliable comparison with clinical data, it is advisable to investigate therapeutic effects following chronic treatment in rodents [128]. It is important to refer that reduction of immobility is interpreted as an antidepressant effect, if it does not increase general locomotor activity, which could be interpreted as a false positive result [123]. This is related with the fact that antidepressants and psychostimulants, respectively, reduce and increase the locomotor activity of rodents in new environments [123]. Moreover, behavioral experiments at different times of the day strongly affect the obtained results [130]. The FST experiments display different results if performed during the dark or light phase, since rodents are more active during the dark phase [131]. Kelliher et al. noticed that rats were more agitated or worsened when taken from the swim apparatus during light phase [131].

Seasonal variations seem to influence the efficacy of antidepressants. In rats, FST revealed significant seasonal fluctuations of the antidepressant effect for TCAs, namely amineptine, amitriptyline, desipramine, imipramine, and mianserin [132]. Maximal reduction of immobility in FST was observed in March for all aforementioned antidepressants. The desipramine and mianserin anti-immobility effect was also evaluated in a light–dark cycle and was not circadian-dependent (Table 3) [132]. Nomifensine, a NE-dopamine reuptake inhibitor, was equally effective throughout the year with no seasonal variations, but it revealed a circadian-dependent effect, more pronounced during the light phase (peak at ZT7) (Table 3) [132]. The interpretation of these results is complicated by the low number of experimental replicates (*n* = 1 or 2). Furthermore, the controlled conditions of rats excluded the influence off the light–dark cycle, humidity and temperature variations that occur during the seasons. Therefore, the observed seasonal effect may be linked with internal mechanisms, namely the daily amplitude of subtype receptor 5-HT_1A_ binding in the rat brain, which is higher in March than December [133].

**Figure 3:**
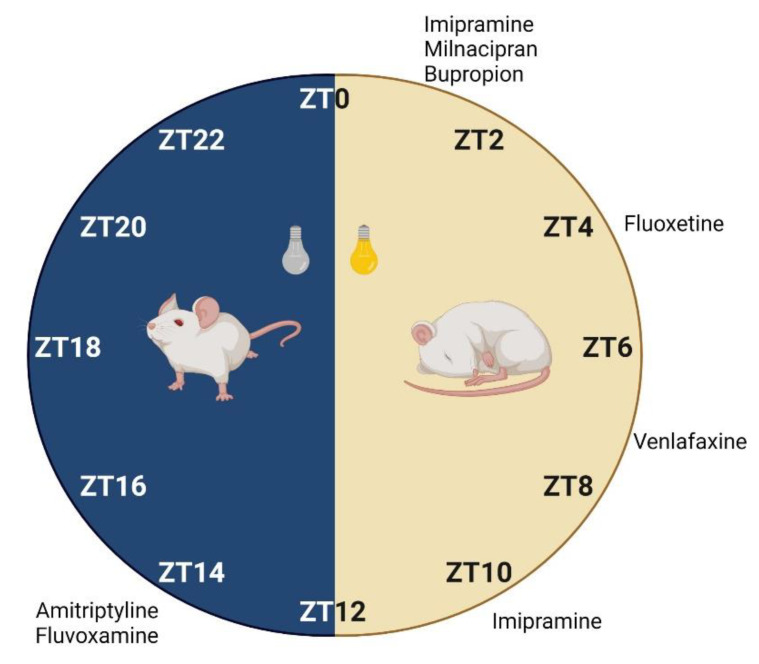
Proposed drug dosing-time of antidepressants according to chronopharmacological studies in rodents. This placement was based on studies performed in mice or rats when higher antidepressant effect was observed during forced swimming [36,104,105] or tail suspension [134] tests. ZT0 represents lights on and ZT12 indicates lights off.

The effect of light–dark cycles on pharmacodynamic studies with antidepressants has also been investigated. In the modified FST, the immobility time of mice treated with SSRI fluvoxamine showed a significant 24 h rhythm, with the lowest immobility at the beginning of the dark phase (ZT14) (Figure 3) without increasing locomotor activity (Table 3) [105]. Moreover, when FST was performed, plasma and brain drug concentrations at 30 min post-intraperitoneal did not exhibit significant differences between ZT2 and ZT14 [105]. Nevertheless, 1 h after administration, plasma concentrations were 1.6-fold higher at ZT2 than ZT14, indicating that pharmacokinetics could play a key role in the acute treatment with fluvoxamine [105]. Another SSRI, fluoxetine, induced a relatively strong oscillation of the antidepressant effect within 24 h, with high amplitudes (39% of mean values) in TST [134]. A maximal effect on the reduction of locomotor activity was observed at the start of the rest-phase (ZT1) with a peak at ZT3.9 (Table 3) [134]. Although not statistically significant, plasma and brain levels of fluoxetine were slightly higher in the morning than evening, which may be related with a higher intestinal absorption in the morning. The results of these studies revealed that both fluvoxamine and fluoxetine have mutual targets but different chronopharmacological profiles. This highlights the importance of performing time-dosing studies with antidepressants.

Dual-action antidepressants may also have different chronopharmacological profiles. In FST, the immobility time of mice treated with TCA amitriptyline exhibited a 24 h rhythm variation with a peak of antidepressant effect at ZT14 (Table 3 and Figure 3), identical to the previously mentioned results for fluvoxamine [105]. This similarity may be associated with the 8-fold higher potency of amitriptyline on SERT rather than NET [110]. On the other hand, milnacipran, a SNRI, was described to reduce immobility and increase swimming at ZT1 rather than ZT13, in FST [104]. Interestingly, climbing was largely superior at ZT13 compared to ZT1. There were no differences of milnacipran levels between dosing times in plasma or the brain (Table 3). Thus, data indicate that SERT inhibition of milnacipran is higher in the morning (swimming), whereas NET is strongly blocked in the evening (climbing) [104].

Chronopharmacodynamic studies with imipramine yielded conflicting results. In rats, a single dose administration of imipramine (30 mg/kg) showed lower immobility and higher climbing in FST in the morning (ZT1) but not evening (ZT13), compared with the control group (Table 3) [36]. The results suggest that a higher concentration may be necessary to achieve an antidepressant effect during evening administrations. Plasma concentrations of imipramine and active metabolite desipramine were 17–37% higher at ZT1 than at ZT13. For this reason, the authors proposed that CYP2D9 peak levels were responsible for lower concentrations during the dark phase [95]. Moreover, the inhibitory action of imipramine is 27 times more potent on SERT than NET, while desipramine is 21 times more potent on NET than SERT [110]. Since an increase of climbing in FST is related to NE reuptake inhibition, desipramine seems to have a more dominant effect on the results of this study. However, in mice, the lowest locomotor activity in TST, under the same conditions, was obtained at ZT13 (calculated peak at ZT10.2) (Table 3 and Figure 3) [134]. Although no significant differences were found in plasma after acute administration [134], this data may be positively associated with circadian rhythm impact on pharmacokinetic parameters of imipramine and desipramine in the brain, with higher accumulation at ZT13 [49], as mentioned in Section 2.1.2. To analyze the discrepancy between these experiments, several factors that may impact the results need to be considered. Firstly, the use of different species (mice and rats) with contrasting chronobiological parameters [135]. Additionally, mice were evaluated at four dosing times (ZT1, ZT7, ZT13, ZT19), while rats were compared only at two dosing times (ZT1, ZT13) which may lead to less reliable conclusions. Lastly, different assays of antidepressant effect were performed, with distinct sensitivities.

Similarly to single dose, chronic treatment with imipramine for two weeks was only effective in the morning, even with doses that were previously inefficient (10 mg/kg) (Table 3) [36]. Only chronic treatment with higher doses revealed an efficient result at ZT13. Chronic treatment with TCAs is linked with a downregulation of cortical presynaptic inhibitory α_2_-adrenoceptors in the rat brain, more evident at ZT1 than ZT13 [36,136]. These adrenoceptors, when localized in presynaptic neurons, reduce NE release in negative feedback [114]. Therefore, the increase of antidepressant effect in the light phase may be associated with a reduction of the expression of α_2_-adrenoceptors that increases NE neural transmission.

The SNRI venlafaxine induced maximal antidepressant effects in the light phase with better results at ZT7 (Table 3) and a calculated peak at ZT9 [134] are shown. It revealed weak circadian rhythms with low amplitude values (29% of mean values) and the plasma and brain levels did not correlate with effect variations. Hence, drug exposure may not be the factor responsible for circadian observations. Meanwhile, bupropion did not reveal daily oscillations of plasma concentration levels, although maximal antidepressant effects had been reached at ZT1 (Table 3) [134]. Locomotor activity increased and was maintained during the light–dark cycle, indicative of its stimulatory motor effects [137].

In general, data from the animal studies herein discussed demonstrate that antidepressants display distinct chronopharmacodynamic outcomes (Figure 3) which should be specifically evaluated. Other behavioral experiments (sucrose preference test or elevated plus maze) could be additionally performed to increase result reliability [138]. Moreover, the use of depressive mice and the monitorization of circadian rhythms could facilitate the implementation of clinical studies in humans. Transferability of data from mice to humans regarding circadian rhythms is challenging. For instance, although mice are nocturnally active and humans are diurnally active, both secrete melatonin during the nighttime [139]. Therefore, the translation of chronopharmacological results of antidepressants from mice to humans needs particular care.

#### 3.1.2. Human Data

**Figure 4:**
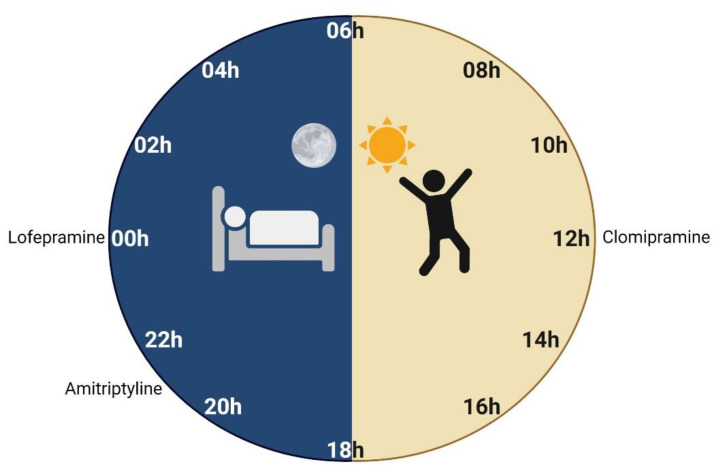
Drug dosing-time of antidepressant drugs according to chronopharmacodynamic studies in humans. This figure includes an optimal time for administration based on lower side effects for amitriptyline [37] and higher antidepressant effects for clomipramine [140] and lofepramine [141].

Despite having yielded interesting and helpful results, chronopharmacodynamic studies in humans have not been performed in recent years. Side effects of TCAs are the principal focus of these types of studies and experiments showed diverse results for different TCAs (Figure 4).

The side effects of amitriptyline seem to be higher after morning administrations. Its antimuscarinic effect, measured through the mean percent decrease from the pre-drug level in salivary flow, demonstrated to be higher if the drug is administered in the morning than in the evening, at 2 h (78 ± 3% vs 59 ± 7%) and 3 h (76 ± 4% and 65 ± 5%) post-administration [37]. Identically, amitriptyline-induced sedative effects, such as drowsiness, confusion, and mental slowness, measured by self-rating scales, were higher with morning than evening doses (Table 4) [37]. These results are in accordance with the pharmacokinetic parameters of amitriptyline mentioned in Section 2.1.1 and Table 2, which presented a faster gastrointestinal Ka in the morning. Although no significant differences of plasma concentration levels or systemic exposure were found at different dosing-times [37], studies in mice demonstrated lower depressive symptoms in the evening [105]. For these reasons, amitriptyline dosing may be recommended in the evening rather than in the morning, given that lower side effects are obtained without compromising its therapeutic efficacy (Figure 4). Nonetheless, additional chronopharmacodynamic studies in humans should be performed to support these observations.

A double-blind chronopharmacological study with TCA clomipramine was performed in 30 patients with MDD for 4 weeks. Single doses of 150 mg/day were administered at 3 different moments: morning, noon, and before bedtime (Table 4) [140]. The highest antidepressant effect was verified at noon (12h20) (Figure 4). Significantly fewer side effects, namely tremors and mouth dryness, were observed at this time, compared with the group in which administration was performed before bedtime [140].

Circadian fluctuations of antidepressant effect were detected for TCA lofepramine, an imipramine derivative [141]. A pilot study was conducted in 30 patients diagnosed with MDD and drug administration was performed at three different times of the day (8 h, 16 h, and 24 h). A single dose of 210 mg lofepramine administered at midnight was significantly more effective than a morning or afternoon dose (Table 4). In addition, a midnight single dose showed better therapeutic results than a 70 mg dose divided three times a day [141]. Based on these results, midnight dosing was suggested as the most favorable administration time (Figure 4). However, it is important to ensure that patient compliance to this posology is maintained. This study highlights that circadian rhythm has a bigger impact than steady-state blood drug levels for an optimal effect of lofepramine. The variable results obtained in the chronopharmacological studies may be associated with the desynchronization of circadian rhythms in depressed patients [142]. This confirms the importance of individualizing chronotherapy to improve the efficacy of antidepressants and reduce their side effects.

### 3.2. Antidepressant Effects on Circadian Rhythms

Circadian gene polymorphisms have been associated with affective disorders, including depression, through the modulation of MAO-A and dopamine neurotransmission [143]. Moreover, pineal abnormalities lead to altered melatonin secretion and circadian disruptions, which are related with clinical subtypes of MDD and its symptomatology [144]. Therefore, the evaluation of circadian rhythm differences in depressed-like mice before and after antidepressant treatments is of utmost importance. Depressed patients experience a wide range of circadian rhythms and sleep-cycle disruptions, and chronotherapy has proved to reduce their depressive symptoms [145]. Therefore, drugs targeted to normalize circadian rhythms could be of interest for the treatment of depression. The main implications of antidepressants on circadian rhythms in pre-clinical and clinical studies are depicted in Table 5.

Several antidepressants enhance plasma melatonin levels, restoring circadian rhythmicity in depression [149,153,154,158,159]. Fluvoxamine increased the plasma levels of melatonin and cortisol in healthy men [153,154], while SSRI fluoxetine and SNRI duloxetine increased 6-sulfatoxymelatonin in depressed patients [149]. Chronic treatment with imipramine or desipramine also resulted in higher peak levels of melatonin in depressed patients [158,159]. Still, imipramine had no significant effect on circadian rhythms of golden hamsters after a 6 h advance in the light–dark cycle [161]. In contrast, desipramine restored photic entrainment of activity, which was previously altered by prenatal exposure to glucocorticoids [160]. Indeed, these mice expressed non-synchronized clock genes in the hippocampus with the light–dark cycle [160]. The results suggest that desipramine promoted glucocorticoid receptor-mediated signaling by upregulating their expression and restoring the synchronization of peripheral clocks in the SCN [160].

The SSRI antidepressants seem to modulate circadian rhythmicity and sleep–wake cycles through the suppression of SERT activity (Table 5) [146]. In vitro, sertraline, fluoxetine, fluvoxamine, citalopram, and paroxetine shorten the period of *Per1*-induced rhythms in rat-1 fibroblasts [146]. This reduction seems to be related to a stronger affinity to SERT [146]. Moreover, subtypes of 5-HT post-synaptic receptors can create a photic (5-HT_2C_ and 5-HT_3_) or non-photic (5-HT_1A_ possibly with co-activation of 5-HT_7_) effect on the SCN, influencing the master clock [150]. Rats administered with fluoxetine showed a non-photic effect by producing shifting during the light phase and attenuating photic resetting during the dark phase [150]. This indicates higher 5-HT_1A_ activity than other subtypes in these rodents, probably because 5-HT_1A_ is expressed predominantly in the cerebellum of adult rats [178]. Sprouse et al. evaluated the regulation of the circadian biological clock through SCN firing in neurons maintained in slice culture [151]. In the presence of L-tryptophan, which maintains the production of endogenous 5-HT in vitro, the addition of fluoxetine induced light-phase advances of SCN firing [151]. In another study resorting to mice with depression-like behavior, *Per2* mRNA levels and circadian period length were restored to normal values after a chronic fluoxetine treatment [152]. The daily rhythms of clock genes and neuropeptide circadian markers were investigated before and after chronic treatment with escitalopram for 8 weeks in depressed patients [147]. Although depressive symptoms decreased, disruption of several daily rhythms (i.e., PER1, CRY, melatonin, cortisol) persisted after treatment [147]. Nevertheless, escitalopram restored daily rhythms of PER2 and BMAL1, as well as the baseline serum melatonin levels [147].

Regarding sleep modulation, an acute citalopram administration to healthy individuals at the beginning of light exposure, increased melatonin suppression in 47% compared to the placebo [148]. In addition, citalopram delayed internal clock rhythms, determined by a dim light melatonin onset (DLMO) in normal lighting conditions, suggesting a decrease of the sleep signal in the evening [148]. Moreover, fluoxetine decreased the response of mice to light-induced phase-delays, similarly to sleep deprivation [179]. On the other hand, vortioxetine or paroxetine treatment revealed a delay of REM onset and a reduction of total sleep and REM time sleep, associated with a higher SERT blockade [157]. Vortioxetine is a multimodal antidepressant and acts as a 5-HT_3_, 5-HT_7_, and 5-HT_1D_ receptor antagonist. It is also a 5-HT_1B_ receptor partial agonist, 5-HT_1A_ receptor agonist, and SERT inhibitor [180]. Both vortioxetine and paroxetine also significantly increased the first stage of the sleep-cycle, related with the transition from wakefulness to sleep [157]. Conversely, paroxetine treatment may induce “hypersomnia” in less than 20% of depressed patients, defined as severe sleepiness and excess sleep duration [156]. Depressed patients who presented paroxetine-induced “hypersomnia” revealed a faster clinical response than patients without this side effect [156]. The results may be associated with a reduction of depressive symptoms caused by insomnia and, therefore, this effect could be beneficial from a pharmacological perspective [156]. In depressed patients with insomnia, an 8-week treatment with fluvoxamine improved sleep parameters and ameliorated insomnia complaints [155]. Additionally, mirtazapine, a noradrenergic and specific serotoninergic antidepressant, improved the sleep continuity of depressed patients following treatment for only 2 days [177]. After 4 weeks, mirtazapine increased slow-wave sleep as well as melatonin plasma levels, and restored sleep-related cortisol secretion. These results may be associated with the antagonist role of mirtazapine on pre-synaptic α_2_-receptors, post-synaptic 5-HT_2_ and 5-HT_3_ receptors, and histamine H_1_, a receptor which induces a sedative effect [177].

The sleep-cycle dysregulation seems to be differently linked with the severity of depression in men and women. In depressed women, disease severity was positively correlated with a low phase angle difference (hour interval between midsleep and DLMO) [142,181]. However, in men, a high phase angle difference was associated with more severe depressive symptoms [181]. Moreover, women who experienced a phase delay after 2 weeks of fluoxetine treatment revealed a poorer pharmacological response after 8 weeks, demonstrating the clinical importance of sex difference and sleep–wake cycle disruption on depression remission [181].

Additionally, MDD has been linked with alterations of neurotrophic factors, specifically brain-derived neurotrophic factor (BDNF), in various brain regions [182]. The BDNF is associated with the regulation of neuronal plasticity and survival, memory, learning, appetite, and sleep [183,184]. In the rat hippocampus, the mRNA expression of BDNF present circadian oscillations, related with plasma corticosterone variation levels [185]. Similarly, in men, plasma BDNF levels show 24 h oscillations [186]. Higher plasma concentrations were detected in the morning than at night and BDNF daily oscillations were positively correlated with cortisol plasma levels [186]. Importantly, women presented BDNF daily oscillations with higher interindividual variability which was not associated with a 24 h period [187]. Regarding MDD, depressed patients demonstrated lower BDNF plasma levels compared with healthy individuals [15]. The increase of BDNF plasma levels and the restoration of the BDNF circadian oscillation has been associated with a positive therapeutic response [188]. It has been demonstrated that antidepressant treatments, such as vortioxetine or agomelatine, increase hippocampal BDNF levels on CMS rats [189,190]. Moreover, chronic antidepressant treatments with fluoxetine, MAOI tranylcypromine, or selective NRI reboxetine, increase neurogenesis in the rat hippocampus [191].

Drug profiles with additional pathways besides the modulation of serotonergic and noradrenergic neurotransmission could be important to develop better antidepressants [145]. For instance, agomelatine is an atypical antidepressant with a unique mechanism of action: it acts both as a melatonergic receptor agonist and a 5-HT_2C_ receptor antagonist [192]. Agomelatine injections increase the 24 h amplitude rhythm of melatonin secretion and the core temperature of rodents, two well-characterized SCN-dependent processes [162]. Like melatonin, agomelatine induces circadian effects on locomotor activity and body temperature directly through the SCN, since a pinealectomy does not alter this ability [163]. In rats, circadian rhythm readjustments induced by oral agomelatine are influenced by its dose (2.5 to 10 mg/kg) and strongly related with its plasma concentrations [193]. Pitrosky et al. observed that rats under constant darkness required a prolonged subcutaneous infusion of 0.25 mg/kg/h for 8 h or 0.5 mg/kg for 1 h to start a resynchronization [194]. Meanwhile, Redman et al. attempted to achieve a complete re-entrainment of rat activity rhythms after an 8 h phase advance of the light–dark cycle [164]. For this purpose, chronic daily subcutaneous injections of agomelatine (1 or 3 mg/kg) were applied, at the pre-shift dark onset [164]. Rodents required about 18 days to be fully resynchronized to the new light–dark cycle, identically to what was observed with melatonin [164]. The aforementioned data demonstrate the strong effect of agomelatine in rodents at resetting circadian rhythms when disrupted (Table 5). Likewise, in healthy individuals, agomelatine induced circadian alterations of cortisol and melatonin levels, core body temperature, and heart rate [165,166].

Sleep–wake states can also be modulated by agomelatine [167,168,169]. Acute administration of melatonin or agomelatine (5 mg) 5 h before bedtime increased rapid eye movement (REM) sleep and advanced sleep–wake cycles in healthy men [167]. Interestingly, when rats were orally treated with agomelatine (10 and 40 mg/kg) before the light phase, no relevant sleep–wake alterations were found [168]. However, after a similar treatment before night phase, Descamps et al. observed unique alterations of sleep–wake states (higher REM and sleep-wave sleep, and lower waking state) [168]. A different sleep profile was obtained from melatonin or 5-HT_2C_ antagonist administrations, suggesting that agomelatine influences the sleep architecture by modulating both melatonergic and 5-HT_2C_ receptors [168]. Still, the involvement of melatonin receptors on depression treatment seems to be time-dependent [195]. Papp et al. administered agomelatine or melatonin (both at 10 and 50 mg/kg) to CMS mice, a validated animal model of depression. Following 5 weeks of treatments performed in the morning or evening, sucrose consumption in the glucose preference test increased, suggesting that animals were less depressed [195,196]. While agomelatine presented similar efficacy after morning or evening treatment, melatonin did not elicit antidepressant-like activity after morning administration [195]. In the presence of melatonergic antagonist S22153, agomelatine efficacy was inhibited in the evening but not in the morning. This suggests distinct mechanisms of action for this drug at different times of administration [195].

To evaluate the resynchronizing properties of agomelatine in depression, psychosocially stressed male tree shrews, an established preclinical model of depression, were treated for 4 weeks with 40 mg/kg/day [170]. Agomelatine reverted the stress-induced nocturnal hyperthermia after treatment, in opposition to other studied compounds (melatonin, fluoxetine, or 5-HT_2C_ antagonist S32006) [170]. Similarly, in mice with a depressive-like phenotype, agomelatine, but not fluoxetine, normalized circadian activity disrupted by chronic corticosterone administrations [171]. In addition, chronic oral treatment with agomelatine in adult rats restored the sleep–wake cycle and the circadian running wheel activity that had been previously disrupted by prenatal restrained stress [169]. In addition, Barden et al. chronically treated a transgenic depressed mouse model with 10 mg/kg of agomelatine to evaluate circadian changes [172]. Readjustments of circadian cycles of core body temperature and locomotor activity were observed following an induced phase shift with agomelatine, but not with desipramine [172].

Clinical studies indicate that agomelatine has antidepressant effects by resynchronizing the circadian rhythms of depressed patients (Table 5) [173,174]. In MDD patients, 25 mg of agomelatine per day increased sleep efficiency, awake time after sleep onset and total amount of slow-wave sleep after 6 weeks [174]. Kasper et al. observed an improvement of sleep and daytime functioning after 1 week of treatment with agomelatine in depressed patients [173]. Compared with sertraline, agomelatine significantly corrected the sleep–wake cycle and decreased depression symptoms quickly, while maintaining a good tolerability profile [173].

As previously mentioned, depression has been linked with abnormal glutamatergic neurotransmission [197]. Activation of glutamate receptors increases the transcription levels of *Per1* and *Per2* in the SCN and suppresses melatonin levels in the pineal gland [198,199]. Ketamine is a N-methyl d-aspartate (NMDA) glutamate receptor antagonist with confirmed antidepressant effects at subanesthetic doses in depressed patients [200]. For rapid and sustained antidepressant action, ketamine activates mitogen-activated protein kinase (MAPK) signaling, specifically extracellular signal-regulated kinase (ERK) through tropomyosin receptor kinase B (TrkB) [201]. The ERK pathway accelerates the differentiation of doublecortin-positive adult hippocampal neural progenitors into functionally mature neurons within 24 h [201]. In addition, MAPK activation allows the endogenous clock to entrain to 24 h environmental cycles [202]. Inhibition of MAPK induces a depressive-like behavior and blocks the ketamine antidepressant effect in rats during FST [203]. Activation of MAPK signaling has been equally associated with an increase of pro-BDNF expression in specific brain areas (hippocampus and *nucleus accumbens*) [203]. Both ketamine and sleep deprivation treatments showed evidence of the influence of circadian rhythms on the rapid antidepressant response through the ERK/MAK pathway [204].

In vitro studies indicated that ketamine inhibits the CLOCK:BMAL1 function by altering the entrainment of clock genes and reducing the daily amplitude of transcription of several clock genes (*Bmal1, Per2, Cry1*) [175]. Single intravenous treatment of 0.5 mg/kg ketamine hydrochloride in depressed patients increased neuroplasticity and improved their mood and sleep quality [176]. Acute ketamine altered circadian timekeeping (amplitude and timing) leading to an initial weak interaction between sleep homeostasis and circadian processes [176]. However, patients in continuous treatment who develop a strong sleep-circadian interaction are associated with fewer relapses and a better ketamine response [176].

In conclusion, commercially available antidepressants have demonstrated to play a critical role on circadian entrainment. Nevertheless, the links behind the modulation of circadian rhythms by antidepressants still require more investigation. New insights may help the design of better chronopharmacological strategies for the treatment of depression.

## 4. Conclusions

Chronotherapy is known to improve drug efficacy and reduce toxicity. The choice of an appropriate dosing-time for antidepressants is a possible factor of variation in pharmacokinetics and may promote therapeutic effects, while reducing adverse effects. Several factors that can affect the pharmacokinetics and pharmacodynamics of antidepressants are modulated by circadian rhythms, which undermine the comprehension of in vivo and human findings. In spite of increasing scientific evidence emerging in this field, further studies in animals and humans remain necessary to determine pharmacokinetic and pharmacodynamic parameters and understand the best time of administration for different antidepressants.

Exploring the chronopharmacological profiles of each antidepressant is expected to provide a more effective pharmacotherapy. Depressed patients can require different dosing-times for the same antidepressant, indicating that individual chronopharmacological therapy should be the primary tool for effective treatment. Moreover, the readjustment of circadian rhythms by some antidepressants is partially responsible for their effectiveness. Thus, restoring circadian rhythmicity is a valid mechanism to promote the development of rapid and sustained treatments in MDD, as it has been discovered in the recent years.

## Figures and Tables

**Table 1:** Chronopharmacokinetic parameters of antidepressants in animal studies.

Antidepressant or Active Metabolite	Animal Species (Gender)	Daily Dose (mg/kg)	Duration (Days)	Route	Biological Matrix	ZT	Pharmacokinetic Parameters						Ref.
							t_max_ (h)	C_max_ (mg/L)	AUC (mg.h/L)	t_1/2__α_ (h)	t_1/2__β_ (h)	k_a_ (h^−1^)	
Amitriptyline	Wistar rat (male)	64	10	Intragastric	Plasma	ZT4	-	-	5.43	-	3.88	0.96	[48]
						ZT16	-	-	4.70	-	2.90	1.40	
Desipramine	Wistar rat (male)	10	1	Intravenous	Plasma	ZT0.5	0.78	54.5	272	0.18	2.76	-	[49]
						ZT12.5	1.71	47.7	259	0.71	1.90	-	
					Brain	ZT0.5	1.86	1.01 ^1^	6.08 ^2^	0.75	1.08	-	
						ZT12.5	2.25	1.02 ^1^	6.50 ^2^	1.20	0.91	-	
				Intraperitoneal	Brain	ZT0.5	2.32	0.85 ^1^	6.90 ^2^	0.83	3.72	-	
						ZT12.5	1.59	1.80 ^1^	10.69 ^2^	0.53	2.91	-	
Imipramine	Wistar rat (male)	10	1	Intravenous	Plasma	ZT0.5	-	-	482	-	1.08	-	[49]
						ZT12.5	-	-	474	-	0.91	-	
					Brain	ZT0.5	-	-	40.43 ^2^	-	1.40	-	
						ZT12.5	-	-	53.21 ^2^	-	2.83	-	
				Intraperitoneal	Brain	ZT0.5	0.43	2.85 ^1^	6.44 ^2^	0.11	1.23	-	
						ZT12.5	0.40	4.80 ^1^	9.67 ^2^	0.11	1.08	-	

Note: Only average values are presented for pharmacokinetic parameters, and some were converted to uniform units. Abbreviations: AUC, area under the curve; Cl, clearance; C_max_, maximum concentration; k_a_, constant absorption rate; t_1/2α_, distribution half-life time; t_1/2β_, elimination half-life time; t_max_, time to reach the maximum concentration; ZT, *zeitgeber* time. ^1^ For brain is mg/g; ^2^ For brain in mg.h/g.

**Table 2:** Chronopharmacokinetic parameters of antidepressants in human studies.

Antidepressant or Active Metabolite	Subjects	Study Design	Daily Dose (mg)	Duration (Days)	Formulation	Time of Administration	Plasma Pharmacokinetic Parameters							Ref.
							t_max_ (h)	C_max_ (mg/L)	AUC (mg.h/L)	t_1/2β_ (h)	k_el_ (h^−1^)	k_a_ (h^−1^)	MRT (h)	
Amitriptyline	10 healthy subjects (♂), 22–31 years old.	Crossover	50	21	Injectable solution	9h00	3.2 *	96.1	1270	15.7	-	0.36 *	-	[37]
						21h00	4.4 *	72.8	1224	17.2	-	0.25 *	-	
Nortriptyline	10 healthy subjects (♂), 22–30 years old.	Crossover	100	14	Oral formulation: 25 mg capsules	9h00	6.2	32	730	15.0	-	-	-	[50]
						21h00	8.8	31	730	16.0	-	-	-	
Trimipramine	12 healthy subjects (6 ♀, 6 ♂), 22–37 years old.	Crossover	100	15	Oral formulation: 100 mg tablet	8h00	2.5	37.8	362	10.9	-	-	10.8	[46]
						20h00	2.8	39.2	376	9.9	-	-	11.5	
					Oral formulation: solution	8h00	1.5 *	48.2 *	372	9.9	-	-	9.8 *	
						20h00	2.5 *	28.8 *	322	11.1	-	-	11.8 *	
Sertraline	10 healthy subjects (♂), 18–45 years old.	Crossover	100	1	Oral formulation: 100 mg tablet	Morning	7.0	24.5	0.664	20.0	0.0347	-	-	[47]
						Evening	7.3	24.4	0.705	20.8	0.0333	-	-	

Note: Only average values are presented for pharmacokinetic parameters, and some were converted to uniform units. Abbreviations: AUC, area under the curve; C_max_, maximum concentration; k_a_, constant absorption rate; k_el_, constant elimination rate; MRT, mean residence time; t_1/2β_, elimination half-life time; t_max_, time to reach the maximum concentration. * Statistically significant values (* *p* < 0.05).

**Table 3:** Chronopharmacodynamic studies of antidepressant drugs in rodents.

Antidepressant	Species (Gender)	Dose (mg/Kg)	Initial of Experiment after Administration (h)	Route	*Zeitgeber* Time (ZT) Administrations	Pharmacodynamic			Drug Concentration	Ref.
						Test	24 h Rhythm Variation	Observations		
Amitriptyline	ICR mice (male)	15	0.5	Intraperitoneal	ZT2, ZT6, ZT10, ZT14, ZT18, ZT22	FST	Yes	Lowest immobility at ZT14.	-	[105]
Bupropion	C57BL/6 mice (male)	20	1	Intraperitoneal	ZT1, ZT7, ZT13, ZT19	TST	No, but significantly different between ZT	Lowest immobility at ZT1.	No significant differences between dosing times in plasma and brain.	[134]
						Locomotor activity	No	Increased		
Desipramine	CD-COBS rats (male)	20	24, 5 and 1	Intraperitoneal	ZT3, ZT7, ZT11, ZT15, ZT19, ZT23	FST	No	-	-	[132]
Fluoxetine	C57BL/6 mice (male)	30	1	Intraperitoneal	ZT1, ZT7, ZT13, ZT19	TST	Yes	Lowest immobility at ZT1.	No significant differences between dosing times in plasma and brain.	[134]
						Locomotor activity	Yes	Lowest locomotion activity at ZT1		
Fluvoxamine	ICR mice (male)	30	0.5	Intraperitoneal	ZT2, ZT6, ZT10, ZT14, ZT18, ZT22	FST	Yes	Lowest immobility at ZT14.	ZT2 > ZT14 in plasma, significantly different after 1h of drug injection. No differences in brain.	[105]
					ZT2, ZT14	Locomotor activity	No	No effect		
Imipramine	C57BL/6 mice (male)	30	1	Intraperitoneal	ZT1, ZT7, ZT13, ZT19	TST	Yes	Lowest immobility at ZT13.	No significant differences between dosing times in plasma and brain	[134]
						Locomotor activity	No	Reduced		
	Wistar Hannover rats (male)	30	1	Intraperitoneal	ZT1, ZT13	FST	Yes	Lowest immobility and highest climbing at ZT1.	ZT1 > ZT13 for imipramine and desipramine in plasma but not significantly different	[36]
		10 for 2 weeks	1	Intraperitoneal	ZT1, ZT13	FST	Yes	Lowest immobility and highest climbing at ZT1.	-	
		30 for 2 weeks	1	Intraperitoneal	ZT1, ZT13	FST	No	-	-	
Mianserin	CD-COBS rats (male)	15	24, 5 and 1	Intraperitoneal	ZT3, ZT7, ZT11, ZT15, ZT19, ZT23	FST	No	-	-	[132]
Milnacipran	Wistar Hannover rats (male)	60	1	Oral	ZT1, ZT13	FST	Yes	Lowest immobility and highest swimming at ZT1.	No significant differences between dosing times in plasma and brain	[104]
Nomifensine	CD-COBS rats (male)	5	24, 5 and 1	Intraperitoneal	ZT3, ZT7, ZT11, ZT15, ZT19, ZT23	FST	Yes	Lowest immobility at ZT7	-	[132]
Venlafaxine	C57BL/6 mice (male)	30	1	Intraperitoneal	ZT1, ZT7, ZT13, ZT19	TST	Yes	Lowest immobility at ZT7.	No significant differences between dosing times in plasma and brain	[134]
						Locomotor activity	Yes	Lowest locomotion activity at ZT7.		

FST, Forced swimming test; TST, tail suspension test.

**Table 4:** Chrono-pharmacodynamics of orally administered antidepressant drugs in humans.

Antidepressant	Subjects	Study Design	Daily Dose (mg)	Duration (Days)	Time Administrations	Pharmacodynamic			Ref.
						Test	24 h Rhythm Variation	Observations	
Amitriptyline	10 healthy (♂) subjects. Range age: 22–31 years old.	Crossover	50	21	9h00 21h00	Antimuscarinic action (saliva flow) and sedation effect by self-rating scales	Yes	Highest salivary flow and lowest sedative effect at 21h00	[37]
Clomipramine	40 patients with MDD (15 ♀, 25 ♂). Range age: 18–65 years old.	Crossover	150	28	8h20 12h20 20h30	HRSD and BDRS	Yes	Lowest depressive symptoms at 12h20	[140]
Lofepramine	30 patients with MDD (22 ♀, 8 ♂). Range age: 25–60 years old.	Parallel	210	21	8h00 16h00 24h00	HRSD and CSRS	Yes	Lowest depressive symptoms at 24h00	[141]

Beck Depression Rating Scale (BDRS); Clinical Self-Rating Scales (CSRS); HRSD, 17-item Hamilton Rating Scale for Depression; MDD, major depressive disorder.

**Table 5:** Main findings of pre-clinical and clinical studies reporting the influence of different classes of antidepressants on circadian rhythms.

Antidepressant	Pre-Clinical Studies	Clinical Studies	References
SSRI			
Citalopram/escitalopram	‑ Modulates *Per1* oscillation in vitro.	‑ Restores daily rhythms of PER2 and BMAL1 and baseline levels of serum melatonin; ‑ Increases melatonin suppression and delays the internal clock rhythm.	[146,147,148]
Fluoxetine	‑ Modulates *Per1* oscillation in vitro; ‑ Induces non-photic effects in light–dark cycle in mice; ‑ Induces light-phase advances of SCN firing; ‑ Normalizes disrupted circadian locomotor activity and clock gene expression in depressive-like mice; ‑ Decreases the response of mice to light-induced phase-delays.	‑ Increases 6-sulfatoxymelatonin in urine.	[146,149,150,151,152]
Fluvoxamine	‑ Modulates *Per1* oscillation in vitro.	‑ Increases plasma levels of melatonin and cortisol; ‑ Improves sleep parameters and reduces insomnia.	[146,153,154,155]
Paroxetine	‑ Modulates *Per1* oscillation in vitro.	‑ Delays REM onset and reduces REM time sleep; ‑ Increases the changeover time of wakefulness to sleep; ‑ May induce “hypersomnia”.	[146,156,157]
Sertraline	‑ Modulates *Per1* oscillation in vitro.		[146]
SNRI			
Duloxetine		‑ Increases 6-sulfatoxymelatonin in urine.	[149]
TCA			
Desipramine	‑ Restores photic entrainment of activity after exposure to glucocorticoids.	‑ Increases melatonin plasma levels.	[158,159,160]
Imipramine	‑ Does not restore photic entrainment after light shifting.	‑ Increases melatonin plasma levels.	[158,159,161]
Atypical			
Agomelatine	‑ Modulates daily rhythm of melatonin secretion; ‑ Induces circadian effects on locomotor activity and body temperature; ‑ Restores resynchronization of light–dark cycle advances; ‑ Improves sleep parameters (only if taken at night); ‑ Restores circadian rhythm activity in depressive-like rodents.	‑ Induces circadian alterations of cortisol and melatonin levels, core body temperature and heart rate; ‑ Improves sleep parameters; ‑ Resynchronizes the circadian rhythms and sleep parameters of depressed patients.	[162,163,164,165,166,167,168,169,170,171,172,173,174]
Ketamine	‑ Alters the entrainment of clock genes; ‑ Resets main clock in the SNC.	‑ Increases neuroplasticity; ‑ Improves sleep quality.	[175,176]
Mirtazapine		‑ Improves sleep continuity; ‑ Increases slow-wave sleep; ‑ Increase melatonin plasma levels; ‑ Reduces cortisol plasma levels.	[177]
Vortioxetine		‑ Delays REM onset and reduces REM time sleep; ‑ Increases the changeover time of wakefulness to sleep	[157]

SCN, suprachiasmatic nucleus; SNRI, serotonin and norepinephrine reuptake inhibitor; SSRI, selective serotonin reuptake inhibitor; TCA, tricyclic antidepressant.

## Data Availability

Not applicable.
